# The Ageing Process Affects the Antioxidant Defences and the Poly (ADPribosyl)ation Activity in *Cistus Incanus* L. Leaves

**DOI:** 10.3390/antiox8110528

**Published:** 2019-11-06

**Authors:** Carmen Arena, Luca Vitale, Anna Rita Bianchi, Carmela Mistretta, Ermenegilda Vitale, Costantino Parisi, Giulia Guerriero, Vincenzo Magliulo, Anna De Maio

**Affiliations:** 1 Dipartimento di Biologia, Università degli Studi di Napoli Federico II, Via Cinthia, 80126 Napoli, Italy; c.arena@unina.it (C.A.); annarita.bianchi@unina.it (A.R.B.); ermenegilda.vitale@unina.it (E.V.); parisi@unina.it (C.P.); guerrier@unina.it (G.G.); 2 Istituto per i Sistemi Agricoli e Forestali del Mediterraneo (CNR-ISAFoM), Via Patacca 85, 80056 Ercolano (NA), Italy; luca.vitale@cnr.it (L.V.); carmela.mistretta@isafom.cnr.it (C.M.); vincenzo.magliulo@cnr.it (V.M.)

**Keywords:** *Cistus incanus* L., photosynthetic apparatus, antioxidants, poly (ADP-ribose) polymerase (PARP) activity

## Abstract

The ageing process in living organisms is characterised by the accumulation of several deleterious changes occurring in cells and tissues. The increase of reactive oxygen species with the advancement of age is responsible for the oxidative damage to proteins, lipids and DNA, enhancing the risk of diseases. The antioxidant response and the activation of the poly(ADP-ribosyl)ation process represent the first defences activated by organisms at all life stages to counteract damage to cell structures and genomic material. The regulation of poly(ADP ribosyl)ation with age is little known in plants, especially in combination with antioxidant defences modulation. In this study, the relationships between poly (ADP-ribose) polymerase (PARP) activity and enzymatic and non-enzymatic antioxidant pool have been studied together with the photosynthetic apparatus efficiency in the Mediterranean species *Cistus incanus* L., examining leaves at different developmental stages: young, mature and senescent. The photosynthetic performance was evaluated by chlorophyll *a* fluorescence measurement, the total soluble and fat-soluble antioxidant capacity, as well as the activities of enzymes superoxide dismutase (SOD), catalase (CAT), peroxidase (POD) and glutathione-S-transferase (GST), were determined by spectrophotometer, PARP activity was assessed by radioactive labelling. The highest photochemical activity was observed in young leaves, together with the highest GST activity. With the progress of the ageing process, the non-enzymatic antioxidant pool (namely ascorbic acid, α-tocopherol) declined, reaching the lowest value in senescent leaves, whereas PARP activity rose significantly. The overall results indicate that the decline of photosynthetic apparatus efficiency during senescence is due to the reduction of specific defences against oxidative damages, which increase the damages to DNA, as demonstrated by PARP activity rise.

## 1. Introduction

Ageing is a natural process associated with the time-dependent general decline in the physiological function of an organism. It represents a multifactorial phenomenon, including genetic, physiological and biochemical changes related to the natural process of growth, to genetic defects and to the relationship between genotype and environmental conditions [1,2,3].

In plants, leaf ageing is a tightly regulated process with a crucial biological purpose: during senescence, metabolic changes and an ordered degradation of structures occur in cells. The decrease of stomatal conductance and photosynthetic rates in the leaves [4] are followed by the degradation of chlorophyll molecules responsible for changes of the leaf colour [5]. By contrast with animals, evident alterations are not observed in the mitochondria and the nucleus that remain intact until the final stages of leaf senescence. Metabolic changes include the hydrolysis of proteins, lipids, nucleic acids and pigments, that are accumulated during the growth phase [6]. At the cellular level, oxidative stress plays an essential role in the ageing process, which seems to be strongly associated with the changes in the prooxidant/antioxidant balance. The oxidative stress occurs when the reactive oxygen species (ROS) generation is transiently or chronically enhanced, and the antioxidant protection system does not counteract the disturbed physiological condition [7]. ROS are generally short-lived highly reactive molecules, derived from the partial reduction of oxygen; they are endogenously generated from healthy cellular metabolism or produced from exogenous sources, including pesticides, ultraviolet (UV) light, metal ions, smoke, ionizing radiation [8,9,10]. ROS represent a continuous challenge for eukaryotic cells, which may maintain under control their excess to avoid apoptosis, necrosis, autophagy and senescence [11,12,13,14]. The cell has particular defence mechanisms in protecting against ROS excess, including the enzymatic and non-enzymatic antioxidants. The main antioxidant enzymes are superoxide dismutase (SOD), catalase (CAT), peroxidase (POD), glutathione peroxidase (GPX), glutathione reductase (GR), and glutathione S transferase (GST) [15]. The non-enzymatic antioxidant defence system includes ascorbic acid (vitamin C), α-tocopherol (vitamin E), glutathione (GSH) and β-carotene [16]. The capacity of cells to counteract the overproduction of free radicals declines with age. The consequences are cumulative damages to important biological macromolecules, like DNA, proteins, and lipids [17,18]. The DNA may be damaged as single-strand breaks (SSBs), double-strand breaks (DSBs), oxidized bases and cross-linking sites [19,20]. The failure in damage repair leads to the cell surviving with altered genetic information. Alternatively, a severe mutational load may cause cell death [21]. In plants such as in animals, several DNA repair mechanisms have evolved to guarantee the integrity of genetic information. The DNA lesions produced by ROS are mainly restored by the base excision repair pathway [22,23,24]. During the process of base excision repair, two nuclear proteins, the poly(ADPribose) polymerase 1 (PARP-1) and the poly(ADPribose) polymerase 2 (PARP-2), regulate the accessibility of nicked DNA to other repair enzymes [25]. The poly(ADPribosyl)ation(PARylation) process represents one of the first cellular responses to oxidative and other types of DNA damages being “sensors” of DNA damaged and involved in the maintenance of genomic stability [26]. Under normal conditions, PARPS have a shallow basal enzymatic activity, which increases dramatically under conditions of cellular stress [27,28]. PARPs activation induces the synthesis of poly(ADP-ribose) (PAR) from nicotinamide adenine dinucleotide (NAD^+^) and the release of nicotinamide as reaction by-product [29,30]. Defects in DNA repair lead to PARP activation and progressive oxidative DNA damage with ageing [31]. A strong correlation has been found between resistance to different stressors, including oxidative stress and longevity in mammalian cells [32,33]. The highest PARylation levels were in long-lived species, in which similar levels of PARP protein were expressed [32]. Thus, poly(ADP-ribosyl)ation capacity was correlated with the ageing process and species-specific longevity [26,34]. Besides, during senescence, in eukaryotes, the activity, stability and localization of several proteins are affected by different post-translational modifications, such as phosphorylation, glycosylation, ubiquitylation, methylation and acetylation [4]. Histone acetylation, methylation, phosphorylation, and PARylation are known to influence the remodelling of the chromatin structure, regulating DNA replication and accessibility of the transcriptional machinery to it with indirect control of gene expression. Plant PARylation was first described in the 1970s, and since then nine proteins with PARP signature were characterized [35,36,37,38]. Besides performing similar functions to those described for the animal counterparts, plant PARPs [39] are also implicated in response to abiotic and biotic stresses [40,41,42], in stress tolerance [38,43] and in developmental processes [44]. The role of poly(ADPribosyl)ation and its implication in the ageing process has been addressed in several studies concerning animals, but not in plants.

The aim of the present research was to study the modulation of the antioxidant defences (enzymatic and non-enzymatic antioxidant pool) and poly(ADPribosyl)ation in response to leaf development to understand the mechanisms at the basis of age-related prooxidant/antioxidant balance and decline of photosynthetic efficiency. To address this, we measured simultaneously oxidative stress markers, namely total soluble and fat-soluble antioxidant capacity, and enzymatic activity of catalase (CAT), superoxide dismutase (SOD), peroxidase (POD) and glutathione-S-transferase (GST) together with measurements of chlorophyll fluorescence emission and PARP activity in young (10-day-old, Y), mature (30 day old; M) and old (45 day old; S) summer leaves of *Cistus incanus* L. In this species the occurrence of a poly (ADP-ribose) polymerase enzyme of 80 kDa, able to synthesize a poly (ADP-ribose) of about 8–10 ADP-ribose unit was already demonstrated [41]. The Mediterranean perennial shrub used in this study is typical of the Mediterranean maquis ecosystems [45] and, from the physiological point of view, is particularly interesting because during the summertime it develops summer leaves especially suitable to cope with drought condition typical of Mediterranean areas [43,46,47]. These leaves exhibit peculiar traits compared to winter ones appearing on the branches in early spring and remaining until the beginning of the winter; during their long lifespan, these leaves experiment and withstand many environmental constraints. 

## 2. Materials and Methods

### 2.1. Plant Material and Growth Conditions

We conducted the experiment on summer leaves of *Cistus incanus* L. subsp. *incanus*, a seasonally dimorphic species with small xeromorphic leaves (summer habitus) produced by plants from the beginning of May until beginning of June as opposed to large mesomorphic leaves (winter habitus) produced from the end of October until beginning of November [43,45,46].

In early May 2014, 10 healthy plants of *C. incanus*, approximately three years old, were screened in the field (Castelvolturno Nature Reserve, Tyrrhenian coast of southern Italy, Naples) for size and uniformity, excavated from field and immediately transplanted in 15 L pots filled with native soil, transported at Department of Biology of University of Naples Federico II and grown in glasshouse in semi-controlled conditions at a mean temperature of 23 ± 2 °C, 65–70% Relative Humidity (RH), 13 h of photoperiod. In order to avoid any additional stress, plants were watered three times a week with variations depending on the evaporative demand of the environment. At the end of June, after 20 days of acclimation at the glasshouse environmental condition, young (Y—10 days old), mature (M—30 days old) and senescent (S—45 days old) summer leaves were selected for fluorescence emission analyses and determination of enzymatic and non-enzymatic antioxidants and PARP activity.

### 2.2. Chlorophyll *a* Fluorescence Measurement

Chlorophyll *a* fluorescence measurements were performed on attached young (Y), mature (M) and senescent (S) leaves of *C. incanus* using the Mini-Pam fluorometer (Walz, Germany) equipped with a leaf-clip holder (Leaf-Clip Holder 2030-B, Walz, Germany) which allows the simultaneous recording of the incident Photosynthetic Photon Flux Density (PPFD) on the leaf surface and abaxial leaf temperature. The maximal PSII photochemical efficiency (F_v_/F_m_) was measured in early morning on 30 min dark-adapted leaves [48]. For the quantum yield of PSII linear electron transport (Φ_PSII_) determination [49], the fluorescence measurements were conducted at midday under natural environmental conditions of PPFD of 800 ± (20) µmol photons m^−2^ s^−1^ and air temperature of 23 (±2) °C.

### 2.3. Isolation of Nuclei and Western Blotting Analysis

Nuclei were isolated as described in Arena et al. [41]. All operations were performed on ice or at 4 °C. Samples of (Y), (M), and (S) leaves (0.1 g) were homogenized at low speed by an Ultra Turrax T8 homogenizer (IKA-WERKE) for 30–40 s. The buffer of homogenization (buffer A) contained 10 mM Tris-HCl pH 8.0, 1 mM ethylenediaminetetraacetic acid (EDTA), 1 mM egtazic acid (EGTA), 1 mM phenylmethylsulfonyl fluoride (PhMeSO_2_F), 10 mM MgCl_2_, 5 mM 2-mercaptoethanol, 1% Nonidet-P40 (1:4, *w*/*v*), and protease inhibitor cocktail (5–10 µg/mL). The homogenates were filtered through three layers of cheesecloth and centrifuged at 1500 g for 30 min at 4 °C. This last procedure was repeated four times. Finally, the pellets, which represent the nuclear fractions, were resuspended in a small volume of buffer A without NP 40.

Y, M, and S leaf nuclear fractions (20 µg) were analysed on 10% polyacrylamide gels in the presence of 0.1% sodium dodecyl sulphate (SDS) tension-active according to Arena et al. [41]. Gels were stained in 0.1% Coomassie G in 10% acetic acid and 30% methanol. For immunoblotting, the electrophoresed proteins were transferred onto a polyvinylidene fluoride (PVDF) membrane (Biorad) at 200 V for 1.5 h at 4 °C in the same buffer used for the electrophoretic run. Filter was placed in blocking solution, containing 50 mM Tris-HCl buffer, pH 8.0; 150 mM NaCl; 0.5% (*v*/*v*) Tween 20 and 3% gelatine for 1.5 h. Subsequently, the filter was incubated for 2 h at room temperature in the same solution integrated with 0, 3% gelatine, in the presence of commercial polyclonal anti-PARP human antibodies (H-250, Santa Crutz), diluted 1:2000, *v*/*v*. After repeated washings in TBS-Tween 0.5%, the filter was incubated for 1 h, at room temperature, in TBS-Tween 0.5% and 0.3% gelatine, containing anti-rabbit secondary antibodies (Thermo Scientific diluted 1:2000) conjugated with peroxidase. The detection of peroxidase activity was conducted by chemiluminescence, using the kit Super Signal West Dura provided by Pierce. The acquisition and analysis of the images was performed using by Quantity One program in a Chemi-doc apparatus (Bio-Rad).

The same filter was incubated in a stripping buffer containing 62.5 m MTris-HCl (pH 6.8), 2% SDS, a final concentration of 0.1 M 2-mercaptoethanol, for 30 min at 50–60 °C according to the methods [41]. Afterward the filter was washed in TBST and analysed by immunoblotting with anti-poly(ADPR) (anti-PAR) polyclonal primary antibodies (H000085-05-B01, Alexis, 1:1000) and horseradish peroxidase-conjugated goat anti-mouse secondary antibody (Pierce, 1:2000) as previously described. Poly(ADPR) antibody is able to identify polymers of ADPR at the last of 5 units.

### 2.4. Poly (ADP-Ribose) Polymerase (PARP) Assay Activity

The assay to measure the ADP-ribosylating activity was carried outfor 10 min at 25 °C. The nuclear fractions (20 µg of proteins) were incubated in presence of 0.4 mM [^32^P] NAD^+^ (10.000 cpm/nmole) in a 500 mM Tris-HCl buffer, pH 8.0; 50 mM MgCl_2_ and 10 mM DTT (reaction mixture) [50]. The reaction was stopped by adding of ice-cold 20% trichloroacetic acid(*w*/*v*). To isolate the product of the reaction, the mixture was filtered on Millipore filters (HAWPP0001, 0.45 μm) and subjected to various washes using 7% trichloroacetic acid. The radioactivity of insoluble acid material associated with the filter was measured in a liquid phase scintillator (Bechman LS 1701). The enzyme activity is expressed in enzymatic milliunit; 1 mU catalyses the synthesis of 1 nmol ADP-ribose/min, at the optimum of pH and temperature.

### 2.5. Determination of Total Soluble and Fat-Soluble Antioxidant Capacity

Stock solutions of ascorbic acid and α-tocopherol were prepared in water and in ethanol respectively, just before use. The concentrations were determined by spectrophotometer considering the absorption coefficients from the literature. All samples were frozen under liquid nitrogen and ground with pestle and mortar to a fine powder. After the addition of 1 mL/g of solvent (water or ethanol), the suspensions were homogenized, transferred to polypropylene tubes, and shaken for 1 h at room temperature in the dark. The suspensions were then centrifuged at 10,000 g for 15 min and the supernatant collected and kept at 4 °C (first extract). The pellets were resuspended, homogenized in another volume of solvent and centrifuged. Finally, the supernatant was joint with the first extract and kept at 4 °C until determinations. Total soluble and fat-soluble antioxidant capacity of Y, M and S leaves were measured according to Prieto et al. [51]. An aliquot of 0.1 mL of supernatant was combined with 1 mL of reagent solution (0.6 M sulfuric acid, 28 mM sodium phosphate, and 4 mM ammonium molybdate). The soluble extracts were incubated at 95 °C, while the fat-soluble extracts were incubated at 37 °C for 90 min in a water bath under a constant shaking. The absorbance was measured at 695 nm against a blank. The blank solution contained 1 mL of reagent solution and the proper volume of the same solvent used for the sample Water-soluble and fat-soluble antioxidant capacity was calculated using a standard curve of ascorbic acid (extinction coefficient 3.4 ± 0.1 × 103 M^−1^ cm^−1^) and α-tocopherol (extinction coefficient 4 ± 0.1 × 103 M^−1^ cm^−1^) respectively and was expressed as equivalents/g of leaves.

### 2.6. Antioxidant Enzyme Analysis

Catalase (CAT), peroxidase (POD) and superoxide dismutase (SOD) activities were determined in Y, M and S leaves. All operations were conducted in ice-cold. The leaves (1 g) were resuspended and homogenized in 0.1 M phosphate buffer (pH 7.5) containing 0.5 mM EDTA. After centrifugation at 4 °C and 15,000 rpm for 15 min., the supernatants were used to measure the activity of antioxidant enzymes. CAT activity was determined according to Wong and Whitaker [52] and Chance and Maehly [53]. The reaction mixture consisted of 0.1 M phosphate buffer (pH 7.0), 200 mM of H_2_O_2_ and enzymatic extract. The activity assay was performed by adding of 50–150 µL enzymatic extract to reaction mixture (150 µL H_2_O_2_, 2 mL phosphate buffer, and water up to final volume of 3 mL). The decrease of H_2_O_2_ was monitored at 240 nm and quantified by its molar extinction coefficient (36 M^−1^ cm^−1^) [54].

POD (EC 1.11.1.7) activity was determined in according to Yuan and Jiang [55] and Chance and Maehly [53]. The reaction mixture contained 0.1 M of phosphate buffer (pH 7.0), 200 mM H_2_O_2_, 135mM of guaiacol. SOD assay was determined by adding 500–1000 µL of enzymatic extract to the reaction mixture (1.2 mL of phosphate buffer30 µL of H_2_O_2_, 200 µL of guaiacol and water in a total volume of 3 mL). Activity was determined by the increase in absorbance at 420 nm caused to guaiacol oxidation (E = 26.6 mM^−1^ cm^−1^).

SOD (EC 1.15.1.1) activity was assayed according to Sun and Zigman [56] and Khopde et al. [57]. Enzymatic assay (final volume of 3 mL) was conducted by adding a reaction mixture (0.1 M carbonate buffer pH 10.0, 5mM epinephrine pH 2.0) to enzymatic extract. Activity was determined by absorbance increase at 320 nm for 1 min. One unit of SOD activity is expressed as the amount of enzyme required to cause 50% inhibition of epinephrine oxidation under the experimental conditions.

The absorbance increase was recorded at 320 nm for 1 min. The linear portion of the reaction progress curve (product versus time) at 320 nm was calculated (∆ Abs320 min^−1^). The slope was used to determine the rate of epinephrine oxidation, which was calculated in the same conditions but without enzymatic extract. One unit of SOD was defined as the amount of enzyme required to reduce the epinephrine auto-oxidation rate by 50%.

The glutathione S-transferase (GST) activity has been estimated according to Habig et al. [58]. Young (Y), mature (M) and senescent (S) leaf samples (5 g) were homogenized in liquid nitrogen to obtain fine powder and suspended in cold potassium phosphate buffer 0.1 M pH 6.5, EDTA 1 mM. The solution was centrifuged at 4 °C at 7000 g for 30 min; the supernatants were then separated and used for enzyme assays. 25 µL of each sample was added to phosphate-buffered saline (PBS) buffer, containing 1-chloro-2,4-dinitrobenzene (CDNB as substrate for GST) at final concentrations of 1 mM reduced glutathione, 1 mM CDNB and 100 mM potassium phosphate buffer (pH 6.5). The formation of the conjugate complex was monitored by spectrophotometer for 5 min, 25 °C at 340 nm. The enzyme activity was expressed as μM/min/g of fresh tissue.

### 2.7. Statistical Analysis

The software package Sigma-Plot 12.0 (Jandel Scientific, San Rafael, CA, USA) was used for graphical and statistical data processing. Statistically significant differences were checked by one-way analysis of variance (ANOVA) based on a significance level of *p* < 0.05. The normal distribution of data was verified by Shapiro–Wilk and Kolmogorov–Smirnov tests. Percent data were transformed through the arcsine function before statistical analysis. The data are mean ± standard deviation (SD) (*n* = 5).

## 3. Results

### 3.1. Photochemical Apparatus Efficiency

**Figure 1:**
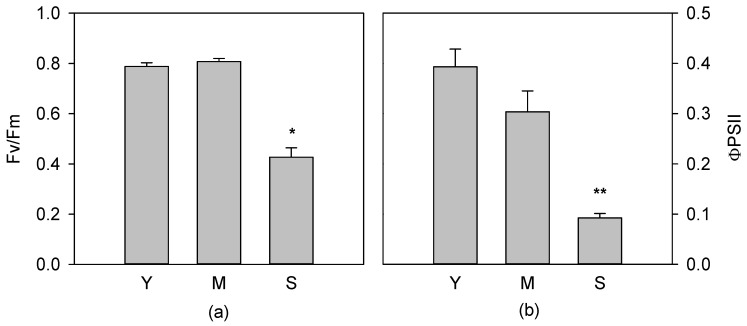
Maximal photochemical efficiency, F_v_/F_m_ (**a**) and quantum yield of linear electron transport, Φ_PSII_ (**b**), of young (Y), mature (M) and senescent (S) leaves of *Cistus incanus* L. Data are mean ± SD (*n* = 5). Results were analysed by one-way analysis of variance (ANOVA) with Holm–Sidak post hoc test. Asterisks represent different levels of significance (** 0.001 < *p* ≤ 0.01, * 0.01 < *p* ≤ 0.05) compared to Y leaves.

The response of photochemical apparatus in *C. incanus* leaves of different age was assessed by chlorophyll fluorescence emission measurements analysing the indexes: maximal PSII photochemical efficiency (F_v_/F_m_) and quantum yield of PSII linear electron transport (Φ_PSII_). As expected, compared to young and mature, senescent leaves showed a strong decrease (*p* < 0.05) of F_v_/F_m_ (Figure 1a) and Φ_PSII_ (Figure 1b). On the contrary, no difference in photochemistry was found between young and mature leaves. 

### 3.2. Poly(ADP)ribosylation Characterization

**Figure 2:**
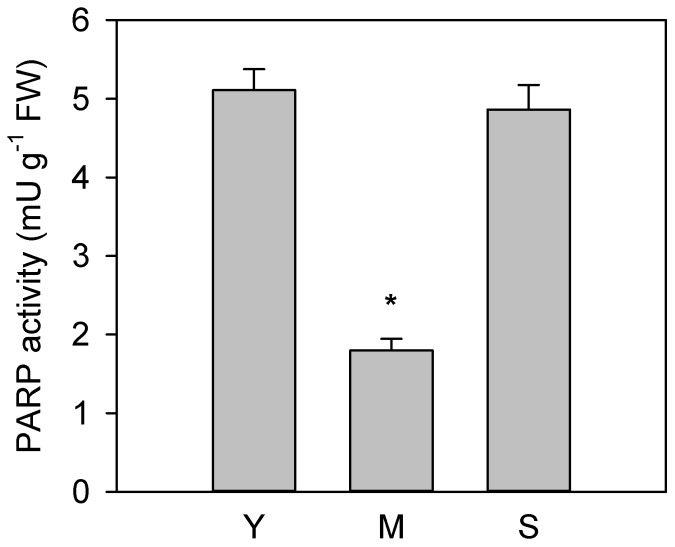
Poly (ADP-ribose) polymerase (PARP) activity measured in young (Y), mature (M) and senescent (S) leaves of *Cistus incanus* L. Data are mean ± SD (*n* = 5). Results were analysed by one-way ANOVA with Holm–Sidak post hoc test. Asterisks represent different levels of significance (* 0.01 < *p* ≤ 0.05) compared to Y leaves.

No significant difference in PARP activity was found between S and Y leaves; on the contrary, PARP activity was three times lower (*p* < 0.01) in M compared to Y and S leaves (Figure 2).

**Figure 3:**
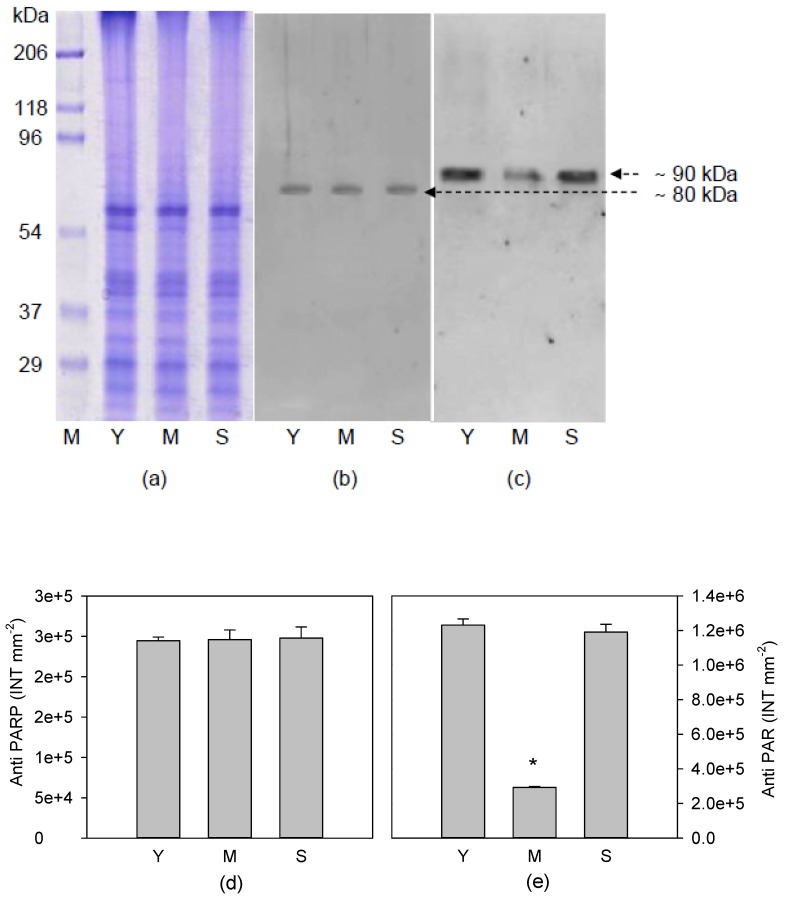
Sodium dodecyl sulfate polyacrylamide gel electrophoresis (SDS-PAGE) 10% (**a**), immunoblotting with anti-PARP (**b**) and immunoblotting with anti-PAR (**c**), in nuclear fractions of young (Y), mature (M) and senescent (S) leaves of *Cistus incanus* L.; densitometric analysis of immunopositive bands to anti-PARP (**d**) and to anti-PAR (**e**). For densitometric analysis data are mean ± SD (*n* = 3). Results were analysed by one-way ANOVA with Holm–Sidak post hoc test. Asterisks represent different levels of significance (* 0.01 < *p* ≤ 0.05) compared to Y leaves.

Moreover, no qualitative and quantitative difference in protein patterns was evidenced (Figure 3a). A single and clear band, corresponding to a protein with molecular weight of about 80 kDa, resulted immunopositive to anti-PARP, which was able to recognize the highly conserved catalytic site of the enzyme (Figure 3b). Western blotting with anti-PAR antibodies used to identify ADPR polymers, consisting of at least five units, showed only one immunopositive signal of about 90 kDa, corresponding to a covalent ADPR protein acceptor (Figure 3c).

Densitometric analysis of bands immunopositive to anti-PARP (Figure 3d) and anti-PAR (Figure 3e) was conducted to correlate the intensity of 80kDa proteins, corresponding to their expression, with poly(ADPR) synthesis. Data showed that the intensity of 80kD protein did not change in examined samples, while the intensity of 90kD protein observed in Y and S leaves was significantly higher than that measured in M leaves (Figure 3e). These results suggest that, although the PARP expression was not affected by leaf age, on the contrary the levels of poly(ADPR) produced in the Y and S leaves were significantly higher than those found in M leaves.

### 3.3. Total Soluble and Fat Soluble Antioxidant Capacity

**Figure 4:**
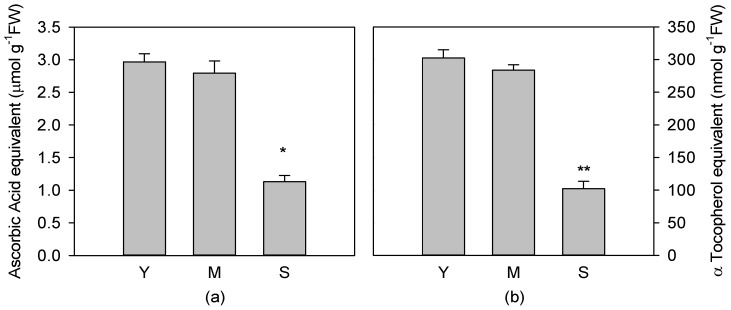
Total soluble antioxidant capacity expressed as ascorbic acid equivalents (**a**) and fat-soluble antioxidant capacity expressed as α tocopherol equivalents (**b**), of young (Y), mature (M) and senescent (S) leaves of *Cistus incanus* L. Data are mean ± SD (*n* = 5). Results were analysed by one-way ANOVA with Holm–Sidak post hoc test. Asterisks represent different levels of significance (** 0.001 < *p* ≤ 0.01, * 0.01 < *p* ≤ 0.05) compared to Y leaves.

Total soluble and fat-soluble antioxidant capacity was evaluated to assess if and how the antioxidant activity may be affected by leaf age. The results showed that in Y and M leaves, soluble (Figure 4a) and fat-soluble (Figure 4b) antioxidant capacities did not show any differences, while in S leaves a significant decrease (*p* < 0.01) of both antioxidant capacities occurred.

### 3.4. Activity of Antioxidant Enzymes

**Figure 5:**
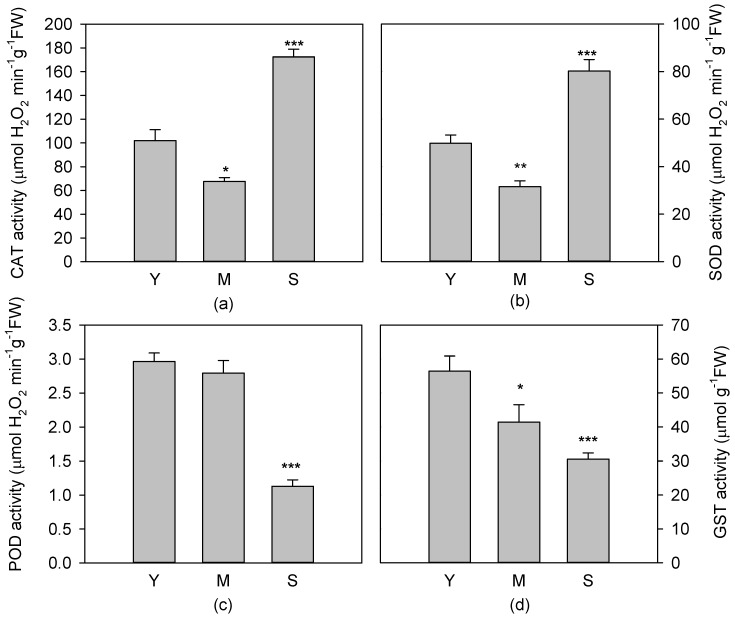
Activity of antioxidant enzymes: catalase, CAT (**a**), superoxide dismutase, SOD (**b**), peroxidase, POD (**c**), and glutathione S-transferase, GST (**d**) in young (Y), mature (M) and senescent (S) leaves *of Cistus incanus* L. Data are mean ± SD (*n* = 5). Results were analysed by one-way ANOVA with Holm–Sidak post hoc test. Asterisks represent different levels of significance (*** 0 < *p* ≤ 0.001, ** 0.001 < *p* ≤ 0.01, * 0.01 < *p* ≤ 0.05) compared to Y leaves.

The activities of catalase (CAT), superoxide dismutase (SOD), peroxidase (POD) and glutathione S-transferase (GST) were analysed to examine particular changes in enzymatic activity related to plant aging. The highest (*p* < 0.01) activity for CAT and SOD enzyme was found in S leaves. A significant difference in CAT and SOD activities was also measured in M compared to Y leaves, with higher values (*p* < 0.05) for Y leaves (Figure 5a,b). No significant variation of POD activity was found between Y and M leaves (Figure 5c). GST activity was statistically higher (*p* < 0.01) in young, compared to mature and senescent leaves. GST activity further decreased (*p* < 0.001) in senescent leaves, reaching the lowest value (Figure 5d).

## 4. Discussion

The ageing process has been associated with an increase of reactive oxygen species (ROS), responsible for cumulative damage to biological macromolecules, including lipids, proteins, and nucleic acids [1,2,3,4]. In the chloroplast, an impairment of photosynthetic membrane functionality leads to the decrease of photosynthetic activity. In fact, with increasing age, the PSII photochemical activity declines indicating a limited capacity of photosynthetic apparatus in the light-harvesting and conversion at reaction centres. Contextually, the increase of PARP activity at the nuclear level may be considered a rapid response of cells to DNA injuries. It has been demonstrated that poly(ADP-ribosyl)ation is one of the most immediate cellular response to genotoxic insults prompted by ionising radiation, alkylating agents, and heavy metals [4,59,60]. 

Numerous studies suggest a link between poly(ADP-ribosyl)ation induced by the DNA damage and ageing/longevity [60,61]; an evident correlation between PARP-1 deficiency and accelerated aging in mammalian cells was also demonstrated [62]. 

In the present work, we demonstrate for the first time that the PARP activity and consequent poly(ADPR) production are strongly influenced by the leaf aging in plants. In plants, three different nuclear PARPs have been previously characterized [38,39,40], and in particular in *Cistus incanus* species a PARP of 80 kDa has already identified [41]. 

The young, mature and senescent leaves of *C. incanus* express only the protein of 80 kDa, recognized by anti-PARP antibodies, specific in the identification of the highly conserved catalytic domain of all three nuclear PARPs. The *Cistus* species used in our experiment express only one of three PARPs documented in plants. We observe a single acceptor of poly(ADPR), corresponding to the same PARP modified with a polymer of about 20 units of ADPR, as indicated by the shift of the molecular weight from 80 kDa to 90 kDa. Therefore, at different leaf growth stages, we do not observe any difference in the length of the synthesized polymer, but only in the amount of poly(ADPR). In particular, the highest levels of poly(ADPR) are found in the Y and S leaves, according to the PARP activity. 

In senescent (S) leaves, the increase of PARP activity as well as of CAT and SOD enzymes indicates that all these scavenger activities co-work against the oxidative stress occurring in cells during ageing. The reduction of the total antioxidant capacity together with the decrease of POD and GST activity suggests the inability of cells to counteract definitely ROS production [63]. However, it is likely that in our study, the oxidative damage to DNA does not lead to cell death because PARP enzymes are hyperactivated guaranteeing a continuous repair of the genomic material. It has been demonstrated in plants that, during senescence, cells are subjected to a degradation of cellular structures, and only the nucleolus and mitochondria remain intact until final stages of leaf senescence [64]; therefore, it is reasonable to suppose that mitochondria could be still functioning ensuring an adequate intracellular NAD^+^ supply for PARP activity. 

At the functional level, ageing determines a reduction of photosynthetic capacity in senescent leaves (F_v_/F_m_ ratio and Φ_PSII_ decreasing), suggesting a loss of functionality of PSII reaction centres. Although it is likely that the decline of photochemistry may be due to a direct effect of ROS on photosynthetic membranes [5], it cannot be excluded that in senescent leaves the photosynthetic electron transport is diverted in the process other than carbon fixation to provide the reductive power for the scavenger enzymes. 

By contrast with S leaves, the high PARP activity found in young (Y) leaves, responsible for the high poly(ADPR) production, seems to be not due to the oxidative DNA damage but rather to other cellular events such as gene expression, transcriptional activity, cell growth, and tissue proliferation [65]. The active growth phase and the high photosynthetic efficiency would guarantee in Y leaves an adequate production of ATP for the synthesis of NAD^+^ for PARP [41]. Moreover, the high total antioxidant capacity, together with the elevated POD and GST activities might assure the apoplastic ROS homeostasis, that control the cell expansion [66]. It is well known that, during the leaf development, a wave of ROS-dependent cell growth sweeps through the leaves [67,68]. Our data suggest that with the progression of ageing, leaves have to carry out a fine-tuning of ROS production and removal adapting to continuous changes of cell metabolism and modulating the antioxidant defences differently [69,70].

## 5. Conclusions

Our work provides the novel insight that the PARP activity and the consequent poly(ADPR) production are strongly influenced by the leaf aging in plants. Leaf aging is associated to oxidative stress due to the ROS increase, responsible for DNA damage and consequent PARP activation. In the senescent leaves, the hyperactivation of PARP activity guarantees a continuous repair of the genomic material avoiding cell death. Overall, this study contributes to a further understanding of physiological, molecular, and biochemical changes occurring in cells during leaf aging and might act as “trailblazer study” for additional research, such as the study of the impact of environmental stressors (e.g., water shortage) on leaf aging.
